# Isojacareubin from the Chinese Herb *Hypericum japonicum*: Potent Antibacterial and Synergistic Effects on Clinical Methicillin-Resistant *Staphylococcus aureus* (MRSA)

**DOI:** 10.3390/ijms13078210

**Published:** 2012-07-03

**Authors:** Guo-Ying Zuo, Jing An, Jun Han, Yun-Ling Zhang, Gen-Chun Wang, Xiao-Yan Hao, Zhong-Qi Bian

**Affiliations:** 1 Research Center for Natural Medicines, Kunming General Hospital, PLA, Kunming 650032, China; E-Mails: zuoguoying@263.net (G.-Y.Z.); anjingmail@163.com (J.A.); zhangyunling@126.com (Y.-L.Z.); kmwgc12@126.com (G.-C.W.); 2 School of Pharmacy, Guiyang Medical University, Guiyang 550004, Guizhou, China; E-Mail: haoxiaoyan@vip.163.com; 3 School of Basic Medical Sciences, Yunnan Traditional Chinese Medical College, Kunming 650500, China; E-Mail: hanzjn@126.com; 4 Center for Infectious Diseases, Kunming General Hospital, PLA, Kunming 650032, China

**Keywords:** anti-MRSA activity, *Hypericum japonicum*, Isojacareubin, MIC, synergy

## Abstract

Through bioassay-guided fractionation of the extracts from the aerial parts of the Chinese herb *Hypericum japonicum* Thunb. Murray, Isojacareubin (ISJ) was characterized as a potent antibacterial compound against the clinical methicillin-resistant S*taphylococcus aureus* (MRSA). The broth microdilution assay was used to determine the minimal inhibitory concentrations (MICs) and minimal bactericidal concentrations (MBCs) of ISJ alone. The results showed that its MICs/MBCs ranged from 4/16 to 16/64 μg/mL, with the concentrations required to inhibit or kill 50% of the strains (MIC_50_/MBC_50_) at 8/16 μg/mL. Synergistic evaluations of this compound with four conventional antibacterial agents representing different types were performed by the chequerboard and time-kill tests. The chequerboard method showed significant synergy effects when ISJ was combined with Ceftazidime (CAZ), Levofloxacin (LEV) and Ampicillin (AMP), with the values of 50% of the fractional inhibitory concentration indices (FICI_50_) at 0.25, 0.37 and 0.37, respectively. Combined bactericidal activities were also observed in the time-kill dynamic assay. The results showed the ability of ISJ to reduce MRSA viable counts by log_10_CFU/mL at 24 h of incubation at a concentration of 1 × MIC were 1.5 (LEV, additivity), 0.92 (CAZ, indifference) and 0.82 (AMP, indifference), respectively. These *in vitro* anti-MRSA activities of ISJ alone and its synergy with conventional antibacterial agents demonstrated that ISJ enhanced their efficacy, which is of potential use for single and combinatory therapy of patients infected with MRSA.

## 1. Introduction

Presently, the global spread of methicillin-resistant *Staphylococcus aureus* (MRSA) is of great concern in the treatment of *Staphylococcal* infections, since it has quickly acquired resistance to all clinical antibacterial agents. Even a glycopeptides resistant strain, *i.e.*, Vancomycin resistant *S. aureus* (VRSA) has also been reported [1]. MRSA has become the most common cause of infections among many pathogenic bacteria, leading to many life-threatening diseases such as endocarditis, pneumonia and toxin shock syndrome. In our hospital, MRSA could be found in over 80 percent of sputum samples of pneumonia elderly patients in the intensive care unit (ICU). Therefore, the developing of agents with novel modes of action is vital for overcoming this troublesome pathogen. Synergy of photochemicals with antibiotics has been viewed as approaching a new generation of phytopharmaceuticals [2,3]. We are devoting effort to search for novel anti-MRSA agents from plant sources with use in traditional Chinese medicine (TCM) in recent years [4–8].

*Hypericum japonicum* Thunb. ex Murray (Di-er-cao or Tian-ji-huang in Chinese; Guttiferae) was first recorded in *Zhiwu Mingshi Tukao* (or *Illustrated Catalogue of Plants*) which dates back to 1848 [9,10]. This medicinal plant has been used to treat viral hepatitis and other infectious diseases for over one hundred years. Bioassay-guided fractionation of the alcoholic extract of the aerial parts of *Hypericum japonicum* led us to identify an anti-MRSA xanthone Isojacareubin (ISJ). The present report investigates the anti-MRSA activity of this compound alone and its synergistic effects in combination with conventional antibacterial agents.

## 2. Results and Discussion

**Figure 1:**
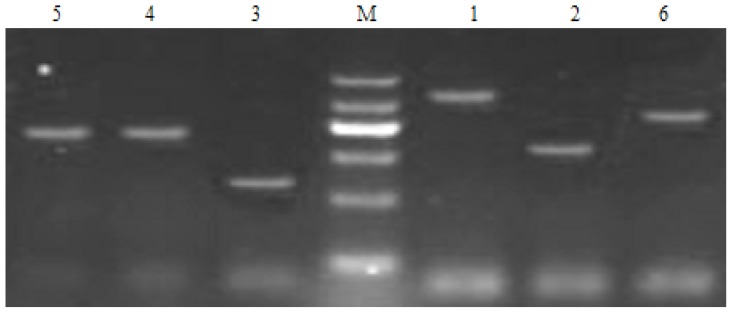
SCCmec III genotyping of methicillin-resistant *Staphylococcus aureus* (MRSA) genes (M: marker, 1: mecA, 2: CCRA1, 3: CCRA2, 4: CCRA3, 5: mec I, 6:IS1272).

**Scheme 1:**
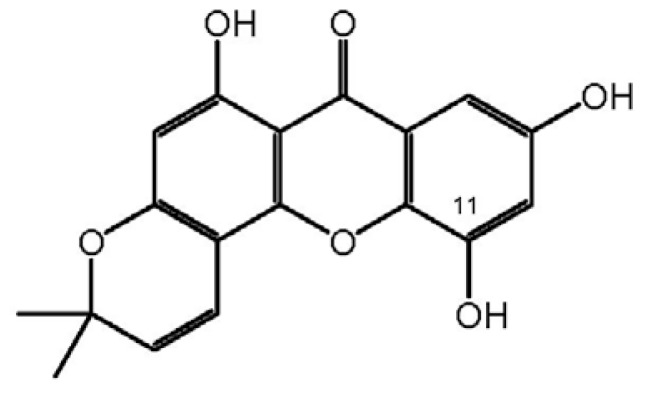
The structure of Isojacareubin (ISJ).

ISJ was isolated through bioassay-guided fractionation of extracts from *H. japonicum* (Table 1) [4]. Its structure was elucidated and identified mainly by spectral analysis and compared with a previous report (Scheme 1) [11]. Ten MRSA isolates, which were used for the evaluation of antibacterial activities, were characterized [4–8], and the presence of the mecA gene and SCCmec genotypes (Figure 1) was demonstrated through multiplex PCR experiments [12].

The *in vitro* anti-MRSA activities of ISJ and four conventional antibacterial agents, *i.e.*, Ampicillin (AMP), Ceftazidime (CAZ), Levofloxacin (LEV) and Azithromycin (AZM) both alone and combined, against the 10 clinical MRSA strains of SCCmec III type [12] are shown in Table 2. The ranges of minimal inhibitory concentrations/minimal bactericidal concentrations (MICs/MBCs, μg/mL) alone were 4~16/16~64 for ISJ, within the order of potency as ISJ (4~16/16~64) = LEV (4~16/8~64) > AMP (16~128/64~512) > CAZ (128~512/128~ >1024) >> AZM (>1024), so the potency of ISJ and LEV were nearly equivalent. In the control experiments, MICs and MBCs of Vancomycin and ISJ against the methicillin-susceptible *S. aureus* (MSSA, ATCC 25923) strain were 1/2 and 8/16 μg/mL, respectively.

Tested by the chequerboard method [13], ISJ showed synergy when it was combined with various antibacterials, with the values of 50% of the fractional inhibitory concentration indices (FICI_50_) at 0.37 (AMP), 0.25 (CAZ) and 0.37 (LEV), respectively. However, the ISJ and AZM combination showed indifference (FICI_50_ = 1.50). The combination of ISJ with CAZ was observed as the most effective, with their values of MIC_50_ (μg/mL) reduced from 8 (ISJ alone) and 512 (CAZ alone) to 1 + 32 (ISJ + CAZ). Combination of ISJ and LEV was observed as less effective, with their values of MIC_50_ (μg/mL) reduced from 8 (ISJ alone) and 16 (LEV alone) to both 2 (ISJ + LEV). According to the CLSI interpretive standard for *Staphylococcus* spp., these values were close to the MICs (μg/mL) of breakpoints of LEV (≤1 for susceptible and ≥4 for resistant) and CAZ (≤8 for susceptible and ≥32 for resistant), respectively, which demonstrated significant potentiation of anti-MRSA effects (or even reversion of the resistance of the corresponding antibacterial agents against MRSA) [14].

**Figure 2:**
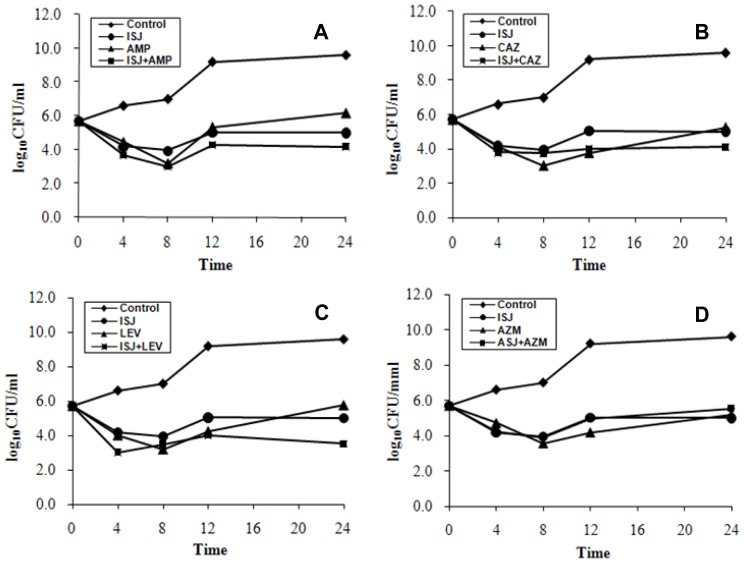
Time-kill curves of the synergistic effect of the combination of Isojacareubin (ISJ) at 1 × MIC concentration with Ampicillin (AMP) (**A**); Ceftazidime (CAZ) (**B**); Levofloxacin (LEV) (**C**); and Azithromycin (AZM) (**D**); against MR004, a clinical MRSA strain of SCCmec III type.

Time-kill curves (Figure 2) showed ISJ alone was the most effective at reducing the viable counts by 4.66 log_10_CFU/mL (−4.66, synergy) of MRSA at 24 h of incubation at the concentration of 1 × MIC, while the counts (log_10_CFU/mL) of combinations of ISJ with AMP (−0.82, indifference), CAZ (−0.92, indifference), LEV (−1.5, additivity) and AZM (+0.53, indifference) were also observed against MR004 (one of the ten isolates) (data not shown). Therefore, the combined bactericidal activities were not as potent as those of bacteriostatic at the concentration of 1 × MIC [15]. Concentrations of higher MIC times of ISJ might produce better combinatory bactericidal effects.

*H. japonicum* is a Chinese herb used in TCM. Its 85% ethanol treated water extract was documented in Chinese Pharmacopoeia as an injection for the treatment of viral hepatitis [16]. In Europe, a plant of the same genus, *i.e.*, *H. perforatum* is known by the name St John’s wort, which is a first-line herbal medicine used for mild to moderate depression [17]. These records demonstrated their safety of therapeutic properties. ISJ is a characteristic constituent in *H. japonicum* and was first found in the plant by Ishiguro *et al*. [11]. Although many xanthone derivatives have been studied for their antimicrobial (including anti-MRSA) activities, the limited distribution in plant resources has meant that ISJ remained as yet to be investigated to the best of our knowledge [18–20]. For the first time we studied its anti-MRSA activities in this report.

As the clinical MRSA which was originally called the “superbug” has been haunting us in recent years and has become an increasingly pressing clinical problem worldwide, anti-MRSA synergistic effects between plant natural compounds and conventional antibacterial agents has further been demonstrated here as a promising way of overcoming current antibiotic resistances [3].

## 3. Experimental Section

### 3.1. Plant Materials

The aerial parts of *H. japonicum* were purchased from the crude drug mart in Kunming, China. They were identified at the Botany Department, Kunming Institute of Botany (KIB), the Chinese Academy of Sciences. The voucher specimen is deposited at the herbarium of KIB.

### 3.2. Bacterial Strains and Media

MRSA strains (ten isolates with SCCmec III genotype) were obtained and characterized from the infectious sputum samples of critically ill patients in Kunming General Hospital [14,21,22]. The presence of mecA gene and SCCmec genotypes were determined by multiplex PCR method (Figure 1) [12]. ATCC 25923 was used as the control strain. Standard Mueller-Hinton agar and broth (MHA and MHB, Tianhe Microbial Agents Co., Hangzhou, China) were used as bacterial culture media. MHB was used for all susceptibility testing and time–kill experiments. Colony counts were determined using MHA plates.

### 3.3. Antibacterial Agents

Four antibacterial agents representing four conventional agents used in clinic were purchased from the manufacturers, *i.e.*, Ampicillin (AMP) (North China pharmaceutical Co., Ltd., Shijiazhuang, China), Ceftazidime (CAZ) (Jida pharmaceutical Co., Ltd., Kunming, China), Azithromycin (AZM) and Levofloxacin (LEV) (Yangzhijiang pharmaceutical Co., Ltd., Taizhou, China). Vancomycin (Eli Lilly Japan K. K., Seishin Laboratories) was used as the positive control agent. Standard Cefoxitin disks were purchased from Tiantan biological products Co., Ltd. (Beijing, China).

### 3.4. Bioassay-Guided Isolation and Identification of ISJ

Powdered aerial parts of *H. japonicum* (4.7 kg) were extracted with 80% ethanol and the extract (500 g) was successively fractionated between water and petroleum ether (P), ethyl acetate (E) and *n*-butanol to afford four fractions. The E fraction (90 g), which showed the most activity by the agar diffusion test described in Section 3.5 below (Table 1) [4], was subjected to column chromatography over silica gel (300–400 mesh; Qingdao, China) and eluted (200 mL/fraction) with ethyl acetate. TLC monitored the eluates and combined to afford three sub-fractions (Frs. A–C_1-6_), gradient elution of Fr. C_2_ with P-E-M (methanol) (4:1:0.2–1:1:0.5) to furnish 20 mg of pure ISJ: light-yellow powder; C_18_H_14_O_6_, ESI-MS: *m*/*z* 327 ([M + 1]^+^); ^1^H NMR (400 MHz) δ: 7.58 (1H, d, *J* = 8.8 Hz, H-8), 7.05 (1H, d, *J* = 10.0 Hz, H-4′), 6.92 (1H, d, J = 8.8 Hz, H-7), 6.15 (1H, s, H-2), 5.72 (1H, d, *J* = 10.0 Hz, H-3′), 1.44 (6H, s, 2′, 2Me); ^13^C NMR (100 MHz) δ: 162.8 (C-1), 98.8 (C-2), 160.2 (C-3), 102.7 (C-4), 51.7 (C-4a), 146.5 (C-4b), 132.9 (C-5), 152.9 (C-6), 115.4 (C-7), 116.6 (C-8), 113.3 (C-8a), 180.4 (C-9), 101.4 (C-9a), 78.6 (C-2′), 127.8 (C-3′), 113.8 (C-4′), 28.3 (C-2′-Me). All the spectral data were identical with the literature [11].

### 3.5. Susceptibility Testing

The inhibition zone diameters (IZDs, mm) of the extracts (30 mg/mL in dimethyl sulfoxide) were determined by the agar diffusion method on MHA plates with inoculums of 1.5 × 10^8^ CFU/mL of the microorganisms following the previous report [5]. MICs/MBCs were determined by standardized broth microdilution techniques with the final inoculums of 5 × 10^5^ CFU/mL in each well of the 96-well plate according to the CLSI guidelines and incubated at 35 °C for 24 h [23,24]. All the experiments were performed in duplicate, with concentrations ranging up to 1024 mg/L for AZM.

### 3.6. Synergy Testing

Potential anti-MRSA synergy was measured by FICIs from the chequerboard method and by time-kill curves as previous report [13]. The FIC of the combination was calculated by dividing the MIC of the ISJ-antibacterial agent combination by the MIC of ISJ or of the antibacterial agent used alone. The FICI was obtained by adding the FIC of the ISJ and that of the antibacterial agent. The FICI results were interpreted as follows: FICI ≤ 0.5, synergy; 0.5 < FICI ≤ 1, additivity; and 1 < FICI < 2, indifference (or no effect) and FICI ≥ 2, antagonism [13,25]. In the killing curves, synergy was defined as ≥2 log_10_ CFU/mL increase in killing at 24 h with the combination, in comparison with the killing by the most active single drug. Additivity was defined as a 1–2 log_10_ CFU/mL increase in kill with the combination in comparison with the most active single agent. Indifference was defined as ±1 log_10_ CFU/mL killing or growth. Combinations that resulted in >1 log_10_ CFU/mL bacterial growth in comparison with the least active single agent were considered to represent antagonism [15,26]. All experiments were performed in triplicate.

## 4. Conclusions

The *in vitro* anti-MRSA activities of Isojacareubin (ISJ) alone and its synergistic effects when used in combination with antibacterial agents demonstrated that ISJ may have potential clinical usage to enhance the efficacy of current antibacterials such as Ceftazidime and Levofloxacin, which warrants further pharmacological studies.

## Figures and Tables

**Table 1:** The screening results of different extraction parts (diameter of inhibition zone: mm)^a^.

Strains^a^	MSSA (ATCC25923)	MRSA 004
Petroleum ether part	29	23
Ethyl acetate part	22	23
Sub-ethyl acetate fraction-A (Fr. A)	17	23
Sub-ethyl acetate fraction-B (Fr. B)	20	15
Sub-ethyl acetate fraction-C (Fr. C_2_)	30	29
*n*-Butanol part	15	17
Water part	15	12
Vancomycin	30	30

a Determined at a concentration of 30.0 mg/mL for the extracts and 1.0 mg/mL for Vancomycin;

b MSSA: methicillin-susceptible *Staphylococcus aureus*; MRSA 004: A strain of methicillinresistant *S. aureus*.

**Table 2:** MICs (/MBCs; μg/mL) and fractional inhibitory concentration indices (FICIs) of Isojacareubin in combination with four conventional antibacterial agents against 10 clinical isolates of SCCmec III type MRSA.

Agents	ISJ^a^	AMP		CAZ		LEV		AZM	
				
	Alone	Alone	Comb^b^	Alone	Comb	Alone	Comb	Alone	Comb
Ranges of MIC (/MBC) (μg/mL)^c^	4(/16)~ 16(/64)	16(/64)~ 128(/512)	4~16 + 1~4	128(/128)~ 512(/ex) + 32~128	32~128 + 1~4	4(/8)~ 16(/64)	1~4 + 1~4	ex	512~1024 + 4~16
MIC_50_^c^	8	64	8 + 4	512	32 + 1	16	2 + 2	-	1024 + 8
MIC_90_	16	128	16 + 4	512	64 + 4	16	4 + 4	-	1024 + 16
Ranges of FICI^d^	-	-	0.37~0.62	-	0.25~0.5	-	0.25~0.5	-	1~1.5
FICI_50_	-	-	0.37	-	0.25	-	0.37	-	1.5
FICI_90_	-	-	0.62	-	0.37	-	0.37	-	1.5
Effect (%)^e^	-	-	ad(30) sn(70)	-	sn(100)	-	sn(100)	-	id(100)

a ISJ, Isojacareubin; AMP, Ampicillin; CAZ, Ceftazidime; LEV, Levofloxacin; AZM, Azithromycin;

b Comb, combined (the binate data are expressed as values of single antibacterial + ISJ);

c MIC_50_, concentration of inhibition against 50% of MRSA strains; MIC_90_, concentration of inhibition against 90% of MRSA strains; ex, concentration of >1024 μg/mL;

d FICI_50_, FICI of inhibition against 50% MRSA strains; FICI_90_, FICI of inhibition against 90% MRSA strains;

e ad, additivity; id, indifference; sn, synergy.
